# Methylation of *avpr1a* in the cortex of wild prairie voles: effects of CpG position and polymorphism

**DOI:** 10.1098/rsos.160646

**Published:** 2017-01-18

**Authors:** M. Okhovat, S. M. Maguire, S. M. Phelps

**Affiliations:** Department of Integrative Biology, University of Texas at Austin, Austin, TX, USA

**Keywords:** *Microtus ochrogaster*, *avpr1a*, retrosplenial cortex, epigenetics, DNA methylation, CpG polymorphism

## Abstract

DNA methylation can cause stable changes in neuronal gene expression, but we know little about its role in individual differences in the wild. In this study, we focus on the vasopressin 1a receptor (*avpr1a*), a gene extensively implicated in vertebrate social behaviour, and explore natural variation in DNA methylation, genetic polymorphism and neuronal gene expression among 30 wild prairie voles (*Microtus ochrogaster*). Examination of CpG density across 8 kb of the locus revealed two distinct CpG islands overlapping promoter and first exon, characterized by few CpG polymorphisms. We used a targeted bisulfite sequencing approach to measure DNA methylation across approximately 3 kb of *avpr1a* in the retrosplenial cortex, a brain region implicated in male space use and sexual fidelity. We find dramatic variation in methylation across the *avrp1a* locus, with pronounced diversity near the exon–intron boundary and in a genetically variable putative enhancer within the intron. Among our wild voles, differences in cortical *avpr1a* expression correlate with DNA methylation in this putative enhancer, but not with the methylation status of the promoter. We also find an unusually high number of polymorphic CpG sites (polyCpGs) in this focal enhancer. One polyCpG within this enhancer (polyCpG 2170) may drive variation in expression either by disrupting transcription factor binding motifs or by changing local DNA methylation and chromatin silencing. Our results contradict some assumptions made within behavioural epigenetics, but are remarkably concordant with genome-wide studies of gene regulation.

## 1. Introduction

Stable and persistent behavioural differences are common among conspecifics, and are thought to contribute to adaptive responses to diverse environments [1–7]. Well-studied examples include the cannibalistic behaviour of spadefoot toads [1], territorial defence of tree lizards [2], anti-predatory responses of snowshoe hares [3] and personality variation among humans [4]. The role that epigenetic factors play in the emergence of such behavioural diversity is an increasingly interesting and active area of work in ecology and evolution, with a variety of studies examining how developmental environments shape the behaviour of adult offspring in the wild [5–7]. As behavioural ecologists seek to explore not only phenotypic variation and its consequences, but also its underlying mechanisms, they have begun to investigate how modifications of chromatin contribute to variation in gene expression and behaviour [6,8]. Of the many known chromatin modifications, DNA methylation at CpG dinucleotides is the most extensively investigated [9]. Despite the exciting prospects for behavioural epigenetics, it remains difficult to follow the relationship between DNA methylation, neuronal gene expression and behaviour in the wild. These difficulties are in part due to the complex regulatory consequences of DNA methylation [10] and our limited understanding of how genetic and epigenetic variation interact to shape brain and behaviour. In this study, we examine how individual differences in sequence and methylation predict neuronal gene expression in the brains of wild prairie voles, *Microtus ochrogaster*.

Traditional studies of DNA methylation focus on CpG sites at a gene's promoter, where CpG methylation often silences gene expression [11]. By contrast, methylation at CpG sites outside the promoter may be associated with either an increase or decrease in expression. For example, methylation within coding sequence can contribute to exon splicing and be associated with elevated expression [12,13]. DNA methylation at more distal elements, such as enhancers and insulators, can either promote or inhibit gene expression [14,15]. Thus, to understand the complex contributions of DNA methylation to gene expression, methylation should be studied across a gene's features. To understand gene regulation in natural settings, it is also critical to consider the genetic variation that could influence methylation and gene expression across these features.

**Figure 1.**
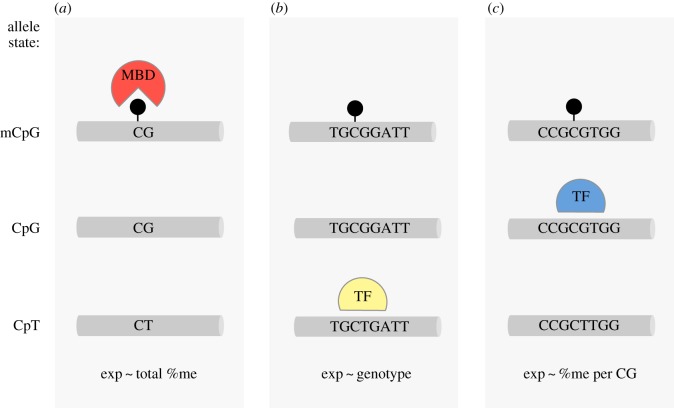
Effects of CpG methylation and polymorphism. Loci with polymorphic CpG sites (polyCpG) can be in several allele states (*left*). The CpG allele can be methylated (*top*, methylation depicted by black circle) or unmethylated (*middle*); but alternative allele (e.g. CpT, *bottom*) is always unmethylated. Depending on the context, these states may have different effects on expression. (*a*) PolyCpGs can change local CpG density and susceptibility to DNA methylation. Methylated CpG allele may facilitate binding of methyl-binding domain (MBD) proteins and change gene expression. In this scenario, the strongest predictor of gene expression is amount of total %DNA methylation at polyCpG site. (*b*) When polyCpG is located at the binding site of a transcription factor (TF) that only recognizes one of the alleles, expression is predicted by genotype at polyCpG. (*c*) If polyCpG is located at the binding site of a methylation-sensitive TF, which only recognizes the CpG allele, expression is influenced by both sequence and methylation status, and is most strongly predicted by the fraction of methylated CpG alleles.

In principle, genetic polymorphism at CpG sites can influence DNA methylation and gene expression by changing either the local density of CpG sites, or by altering specific binding sites for transcription factors (figure 1). Though poorly understood, the overall density of CpG sites seems to be important for shaping the epigenetic status of a regulatory element. Short stretches of densely packed CpGs (approx. 1 kb) known as CpG islands (CPGi) can lead to stable de-methylation [16]. By contrast, regions just outside CpG islands have lower CpG density, exhibit tissue-specific methylation, and are more likely to have single nucleotide polymorphisms (SNPs) within a CpG site [17,18]. A CpG polymorphism—for example, TG/CG or CA/CG—is referred to as a polyCpG. By altering local CpG density, such polymorphisms could change the likelihood of recruiting repressive proteins with methyl-binding domains (figure 1*a*; [11,19]). PolyCpGs may also affect binding of a transcription factor that is sensitive to variation in motif sequence (figure 1*b*), methylation or both (figure 1*c*; [10,11,20]). These alternatives reveal some of the complex ways in which CpG polymorphisms may interact with epigenetic mechanisms to produce differences in developmental sensitivity, plasticity and complex behaviours.

Although DNA methylation is present in a wide range of taxa [21] and CpG polymorphisms are common [18], their contributions to natural neuronal and behavioural diversity are not well understood. Genetically diverse non-model species allow us to apply modern molecular techniques to examine natural variation in genetics and epigenetics, as well as their association with neuronal and behavioural variation. In this study, we use the socially monogamous prairie vole, *M. ochrogaster*, to investigate the interaction between DNA methylation, CpG polymorphism and the expression of the vasopressin 1a receptor (*avpr1a*), a gene critical for social behaviour in this and other species.

Prairie voles are socially monogamous rodents, but approximately 25% of the offspring are sired outside the pair (known as extra-pair fertilizations, [22]). Variation in prairie vole sexual fidelity is predicted by differences in space use that seem to be mediated by variation in *avpr1a* expression in the retrosplenial cortex (RSC-V1aR), a brain region important in spatial memory [23,24]. Among laboratory-reared animals, the cortical expression of *avpr1a* is highly predicted by four SNPs that together define ‘HI’ and ‘LO’ alleles. Interestingly, one of the polymorphisms (SNP 2170) is a polymorphic CpG weakly linked to several other polyCpGs. These polymorphisms occur within a short sequence identified as a putative enhancer by ChIP-seq targeting the histone mark H3K4me1 [24], and its methylation status predicts cortical V1aR abundance among laboratory-reared animals. Among wild-caught animals, we found that the relationship between genotype and phenotype was weaker, and speculated that this was due to increased variation in developmental environment [24]. In this study, we ask whether methylation of the putative intron enhancer is also able to predict cortical expression of *avpr1a* among wild prairie voles. We expand on these findings by investigating sequence variation and methylation across a much broader expanse of the locus, allowing us to more systematically explore how genetic and epigenetic variation contribute to neuronal gene expression in the wild.

We first characterize the *avpr1a* locus by identifying CpG islands and examining the distribution of polyCpGs across the *avpr1a* locus. Next, we validated a sequencing approach to estimate methylation at 122 CpG sites across approximately 3 kb of *avpr1a*, spanning from promoter to the putative intron enhancer. We then use these data to examine the pattern of methylation across *avpr1a* features, to test how methylation in different features predicted cortical *avpr1a* expression, and to ask whether polymorphic CpG sites contribute to CpG density or sequence-specific effects of methylation. In the process, this study explores how previous results from genome-wide studies of methylation inform our understanding of individual differences in brain and behaviour.

## 2. Material and methods

### 2.1. Wild-caught samples and tissue processing

In total, 32 wild adult male (*n* = 18) and female (*n* = 14) prairie wild voles were collected from Champaign County, IL, USA. Brains were frozen immediately on dry ice, stored at −80°C and later sectioned at 20 µm thickness and 100 µm intervals. V1aR autoradiography from these samples has been reported previously, and methodological details are provided there [23,24]. An alternative set of fresh-frozen brain sections was used as a source for genomic DNA in the Sanger sequencing of the locus.

To examine the methylation status of *avpr1a*, we dissected the retrosplenial cortex from a third set of alternative fresh-frozen sections. Fresh-frozen sections were not available for two of 32 animals, which reduced our sample size to 30 individuals. We performed genomic DNA bisulfite conversion using the EpiTect Plus LyseAll Bisulfite Kit (Qiagen), following manufacturer's instructions.

### 2.2. Characterization of the *avpr1a* locus

Sequencing of the *avpr1a* was performed as described previously [24]. Sequence reads were aligned to *avpr1a* reference (AF069304.2, NCBI) in Geneious 5.5.7 software to find fixed and polymorphic CpGs (polyCpGs). PolyCpGs were defined as SNPs occurring at the C or G within a CpG dinucleotide. CpG polymorphisms present in only a single individual were disregarded, as they are too rare to be useful in examining associations.

**Figure 2.**
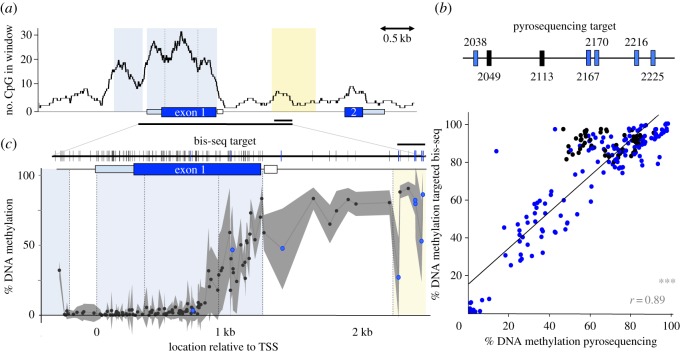
CpG distribution and DNA methylation across the *avpr1a* locus. (*a*) A sliding window (window = 300 bp, step = 1 bp) of CpG count along 8 kb of the *avpr1a* locus. Two predicted CpG islands are shaded light blue and the putative intron enhancer is yellow. The region covered by pyrosequencing (0.2 kb) and bis-seq (3 kb) marked by horizontal black bar. (*b*) Pyrosequencing assay included two fixed CpGs (black) and five polyCpGs (blue) within the putative enhancer. Pyrosequencing methylation measures correlate with bis-seq results (*r* = 0.89, *p* < 0.001). (*c*) *Top,* 113 fixed sites included in bis-seq target are represented by black vertical bars and eight polymorphic CpGs (polyCpGs) are marked blue. *Bottom*, average %DNA methylation from bis-seq at fixed (black) and polymorphic (blue) CpGs across 3 kb of *avpr1a* locus. Standard deviation depicted in grey shading. Gene feature borders are separated by dashed lines. ***p ≤ 0.001.

To characterize CpG density across the locus, we calculated the CpG count in 300 bp sliding windows across the reference *avpr1a* sequence. Also, we predicted the position of CpG islands at the *avpr1a* locus (AF069304.2, NCBI) using the online EMBOSS Cpgplot tool [25]. We used a window size of 300 bp and traditional CpG island algorithm criteria, including an island length more than 200 bp, GC content more than 50% and Obs_CpG_/Exp_CpG_ > 0.60 [26]. Our CpG density analysis revealed two CpG islands, CpGi.1 is 5′ of the transcription start site (TSS) and includes parts of the *avpr1a* promoter, while CpGi.2 includes the first exon. CpGi.2 exhibited a distinct tri-modal pattern in CpG density. To capture this heterogeneity in CpG density, we subdivided CpGi.2 into three compartments defined by local minima in CpG density (figure 2*a*). These features were the basis for the parsing of our analysis of methylation data across 3 kb of the *avpr1a* locus described below.

### 2.3. DNA methylation measurements

#### 2.3.1. Pyrosequencing

We used a nested PCR strategy to produce two pyrosequencing amplicons as described previously [24]. The biotin-labelled PCR amplicons were sent to Epigendx (Hopkinton, MA, USA) for DNA methylation pyrosequencing (assay IDs: Cluster 1FS2 and Cluster 2FS2). DNA methylation at CpG sites was reported as %(unconverted C/ (unconverted C+ converted T)) for each CpG site.

#### 2.3.2. Targeted bisulfite sequencing

To examine individual differences in methylation across major gene features of the *avpr1a* locus, we generated a series of five amplicons spanning approximately 3 kb from the promoter to the intron enhancer.

We used a semi-nested PCR approach to amplify 350 bp upstream of the TSS and the first exon (table 1A). The outer PCR reaction included KAPA HiFi Uracil + mix (KAPA biosystems), 300 nM of each primer (table 1A) and 1.5 µl of bisulfite converted gDNA with the following settings: 3 min at 95°C, (20 s at 98°C, 30 s at 52°C and 90 s at 68°C) × 36. Two following semi-nested inner PCR reactions consisted of HiFi Uracil + mix (KAPA biosystems), 400 nM of each primer and 2 µl of undiluted outer amplicon. Amplifications were performed with the following settings: 2 min at 95°C, (20 s at 98°C, 30 s at 55°C and 90 s at 68°C) × 25.

**Table 1.** PCR primers (5′ → 3′) for bis-seq amplifications.

target	primers for outer PCR	primers for inner PCR (if nested)
A. exon1 + promoter	F1:GAAAYGTTGGGTTTGGTGGATTAGTTAG	F1:AAAYGTTGGGTTTGGTGGATTAGTTAG
	R1:AAAATAATCTTCACRCTACTAACACAAAAC	R2:AATACCCCAAAACTAAATAAAAATAACCCAAC
		F2:GGTTTTGTAGAGGAATTTAGGAGTTTTTTAG
		R1:AAAATAATCTTCACRCTACTAACACAAAAC
B. exon1 − intron boundary	F3:TAGTTTATGGTGGTTTTTGAGYGTTGAG	—
	R3:CTTACACAATAAACTCTAAAACRATTTCTA	
C. intron	F4:GGGGTTTTTGGTTAYGTTTTGTGTTAGTAG	F4:GGGGTTTTTGGTTAYGTTTTGTGTTAGTAG
	R4:CACAAAAATCACCTAAAACCATCCTAAATTTCAA	R5:CCAAAAAAATATATCCATCCCTATCCTTA
		F5:GGGGTTAGGAGTTAGTATGTATGGATTATAT
		R4:CACAAAAATCACCTAAAACCATCCTAAATTTCAA

Using primers provided in table 1B, we amplified a 1.6 kb amplicon around the exon1–intron boundary with PCR composition similar to the reaction described above and the following settings: 3 min at 95°C, (20 s at 98°C, 30 s at 58°C and 90 s at 68°C) × 40.

We used a semi-nested PCR approach to amplify 1.5 kb of the intron in a PCR reaction consisting of KAPA HiFi Uracil+ mix (KAPA biosystems), 300 nM of each primer (table 1C) and 1.5 µl of bisulfite converted gDNA with the following settings: 3 min at 95°C, (20 s at 98°C, 30 s at 52°C and 90 s at 68°C) × 36. Inner PCR reactions consisted of GoTaq Hot Start Colorless Master Mix (Promega), 200 nM of each primer and 1 µl undiluted outer amplicon. Amplifications were performed with the following settings: 3 min at 93°C, (30 s at 93°C, 30 s at 55°C and 90 s at 70°C) × 35, 2 min at 70°C.

All final PCR products were visualized on agarose gel and gel-extracted using Qiagen gel extraction kit (Qiagen).

#### 2.3.3. Library preparation

Following PCR cleanup, DNA concentrations were measured on a Nanodrop 2000 Spectrophotometer (Thermo Fisher Scientific). For each individual, PCR amplicons were mixed in equimolar ratios and brought to a final volume of 500 µl with 1× TE. Sample pools were then sonicated with Q125 sonicator (Qsonica) on ice for 25 cycles (10 s pulse, 10 s rest) at 50% amplitude. DNA was then precipitated with standard EtOH precipitation and eluted in 1× TE.

For each individual, 50 ng of the sheared DNA pool was used to construct Illumina paired-end libraries using the Nextflex ChIP-Seq kit (BioScientific) according to manufacturer's instructions with minor modifications. Briefly, samples were end-repaired and size-selected to capture 300–400 bp fragments. Size-selected fragments were adenylated and barcoded with Nextflex Illumina DNA barcodes (BioScientific). We used the KAPA library amplification kit (KAPA biosystems) to amplify the library for five to six cycles according to manufacturer's protocol. Libraries were sequenced on the MiSeq Illumina platform (2 × 250PE) at UT sequencing core facility (Austin, TX, USA).

#### 2.3.4. Sequence analysis

Reads were shortened to 130 bp by trimming low-quality 5′ ends. Next, we used *Trim-galore!* [27] to remove remaining adaptor contamination, low-quality reads (Phred < 20), short reads (less than 16 bp) and reads with a missing pair. The reference *avpr1a* sequence (AF069304.2, NCBI) was modified to include known SNPs. SNPs that involved CpG sites were left as CpG dinucleotides and the rest of SNPs were replaced by their corresponding ambiguous IUPAC symbol. These modifications allowed us to measure DNA methylation at both fixed and polymorphic CpG sites and avoid allelic bias in alignment. We used *Bismark v0.7.7* [28] with *bowtie2* [29] for read alignment. Next, we used Bismark's *Methylation extractor* tool and a custom python script, to compile counts of methylated and unmethylated reads at each CpG site and determine per cent CpG methylation. We also obtained non-CpG cytosine methylation within CHG and CHH contexts (H is A, C or T) from Bismark alignment reports. All methylation values were exported to R (http://www.r-project.org/) for further analysis.

#### 2.3.5. Defining features of the bisulfite sequencing target

To accommodate potential heterogeneity in methylation across the locus, we used the boundaries of *avpr1a* features defined above to partition our bisulfite-sequencing (bis-seq) target (figure 2*b*). The first approximately 100 bp of our bis-seq target corresponds to the 3′ region of **CpGi.1**. An approximately 200 bp region between the CpG islands includes the TSS and the 5′ 18 bp of the 5′ UTR; we labelled this segment as **Promoter**. The labels **CpGi2a–c** correspond to three local peaks in CpG density within CpGi.2. The label **Intron** refers to an approximately 1 kb sequence from the end of CpGi.2 to the beginning of a putative intron enhancer. Lastly, our bis-seq target overlaps with the first approximately 300 bp of a putative intron **Enhancer** identified by H3K4me1 ChIP-seq on prairie vole retrosplenial cortex [24].

### 2.4. Statistical analysis

#### 2.4.1. Bisulfite sequencing technical validation

We used a linear model to examine the correlation between methylation values obtained at seven intron enhancer CpGs by targeted bis-seq to pyrosequencing data from the same sites. To determine the null distribution of the expected correlation, we randomly assigned pyrosequencing methylation values to individuals 1000 times and each time measured the Pearson correlation coefficient between pyrosequencing and bis-seq values. We used these randomized correlation coefficients to estimate a null distribution and resulting *p*-value.

#### 2.4.2. CpG co-methylation within and between gene features and across *avpr1a*

We used a linear model to examine the relationship between co-methylation (Pearson's correlation coefficient) and distance between the CpG pair within and between gene features. Significance of effects was determined by permutation analysis. We also used a heatmap to visualize heterogeneity in co-methylation between all pairs of CpGs across our bis-seq target.

#### 2.4.3. *Avpr1a* alleles and enhancer CpG differences

We used sequence at the intron enhancer SNP 2170 (T/T, T/G, G/G) to assign HI/HI, HI/LO and LO/LO *avpr1a* genotypes. We scored genotypes with values 0, 1 and 2 corresponding to the number of HI alleles present. We ran ANOVA and Kendall's rank correlation analyses to compare V1aR abundance in the retrosplenial cortex (RSC-V1aR), DNA methylation and enhancer CpG count among *avpr1a* genotypes. Data on genotype association with RSC-V1aR abundance (figure 5*b*) were previously published [24], but are included here for completeness.

#### 2.4.4. PolyCpG frequencies and distribution

We observed 30 polyCpG sites across the locus, one of which had three alternative alleles. For the 29 bi-allelic SNPs, we calculated the number of variants corresponding to each of six possible CpG polymorphisms: CpA, CpC, CpT, ApG, GpG and TpG. We performed a 6 × 2 *χ*^2^ test comparing the observed SNP frequencies to a neutral expectation in which each polymorphism is equally likely.

To examine heterogeneity in the distribution of polyCpGs, we used a two-tailed Fisher's exact test to compare the ratio of polyCpGs:total CpGs within the CpG islands to the ratio at the rest of the locus. Similarly, we compared polyCpGs:total CpGs and polyCpG:nucleotides in the enhancer to the rest of the locus. Fisher's exact and *χ*^2^ tests were performed using the online GraphPad software (available at http://graphpad.com/quickcalcs/contingency1.cfm).

#### 2.4.5. Sequence-specific effects of polyCpGs and methylation

At the eight polyCpG sites included in our bis-seq target, we used linear regression to test the association of RSC-V1aR with *total %DNA methylation* at each polyCpG, *genotype*, and with *%methylation per CpG*—a measure normalized for the number of CpG-containing alleles present at a specific polymorphic site. To be explicit, *total %DNA methylation* is defined as the proportion of reads that carry a methylated CpG at the site of interest, regardless of genotype. *Genotype* is the number of CpGs the individual possesses at a polymorphic site (0, 1 or 2). Lastly, for individuals homozygous for a CpG or alternative allele, *%methylation per CpG* equals *total %DNA methylation,* but for a heterozygous individual, it is 2* (*total %DNA methylation* at CpG site).

To predict transcription factor binding around polyCpG 2170 and to test if sequence differences between HI and LO affect their binding, we used the transcription factor affinity predictor web tool for SNP comparisons (sTRAP, [30]). We used the HI and LO sequence in a 20 bp window centred at polyCpG 2170 and selected transcription factor matrices from TRANSFAC (vertebrates-only) with a mouse-promoter background model. The *p*-values were corrected using Benjamini–Hochberg corrections [31]. Transcription factors that had significant (*p* < 0.05) affinity to at least one of the genotypes at polyCpG 2170 were selected and ranked from highest to lowest genotype difference in affinity. Lastly, we examined the Allen Brain Atlas [32] to examine whether any of the identified transcription factors were expressed in the retrosplenial area of the mouse brain.

## 3. Results

### 3.1. Characterization of the *avpr1a* locus

We sequenced and analysed approximately 8 kb of the *avpr1a* locus in 32 wild-caught prairie voles and found a total of 172 fixed CpG sites and 30 polymorphic CpGs (polyCpGs). We observed that CpG density was not homogeneous across the locus, with evidence of two CpG islands (figure 2*a*). The first predicted CpG island (CpGi.1) is approximately 0.4 kb long and starts approximately 0.6 kb upstream of the *avpr1a* TSS. The second CpG island (CpGi.2) is 1.3 kb long, and encompasses most of the 5′ UTR, all of the first coding sequence and a short region of the intron. CpG density was variable within this CpGi, as evident by three local peaks of CpG density in a sliding-window analysis (figure 2*a*).

The approximately 2 kb of sequence that flanks either side of a CpG island are known as CpG island-shores or CpGi-shores. CpGi-shores have high methylation variation and show tissue-specific differential methylation [17]. At the *avpr1a* locus, the CpGi-shores include a 2 kb region upstream of CpGi.1 and a 2 kb region downstream of CpGi.2, which includes most of the intron and all of a putative intron enhancer identified previously by H3K4me1 ChIP-seq [24]. The CpG density is relatively low within CpGi-shores and in features located outside the shore boundaries (e.g. second exon, figure 2*a*).

### 3.2. DNA methylation measurements and bisulfite sequencing technical validation

To control for tissue differences in methylation, all our methylation measures were obtained directly from RSC dissections of wild-caught brains. While these methylation measures reflect averaged measures across multiple cell types, this approach is much more accurate than measuring methylation in the whole brain or in peripheral proxy tissues, such as blood [33].

We used bisulfite pyrosequencing to measure DNA methylation in the putative intron enhancer of 30 wild-caught animals. Our pyrosequencing assays measured methylation at two fixed and five polymorphic CpG (polyCpG) sites (figure 2*b*). One of the polyCpGs (polyCpG 2170) has previously been shown to be highly predictive of V1aR abundance in the retrosplenial cortex (RSC-V1aR) in prairie voles [24]. Of 30 samples, none failed standard QC measures (threshold for QC rejection: bisulfite conversion efficiency less than 93%) and genotypes were correctly captured at all polyCpG site.

We used a targeted bis-seq approach to expand our DNA methylation measurements. Our bis-seq assay spanned from 300 bp upstream of the *avpr1a* TSS to 2.3 kb downstream, and covered 114 fixed and eight polymorphic CpG sites (figure 2*c*). Our assay generated single CpG resolution methylation measures and 100% coverage of all targeted CpG sites for all 30 wild-caught voles. To assess the accuracy of our bis-seq assay, we compared bis-seq DNA methylation measurements at each of the seven putative enhancer CpG sites to pyrosequencing methylation measures at the same sites. Levels of methylation estimated by targeted sequencing were slightly higher but broadly similar to those we obtained by pyrosequencing. We regressed these measures against one another and found that methylation measurements from the two techniques agree, especially at polyCpG sites (*r* = 0.89, *p* < 0.001; figure 2*b*).

In addition to examining canonical CpG methylation, we used our bis-seq data to examine DNA methylation in CHG and CHH contexts (where H is A, C or T). Non-CpG methylation has previously been found in the mammalian adult brain, where it is negatively correlated with expression [34]. Based on our bis-seq data, however, %CHH and CHG methylation at the *avpr1a* locus were both very low (CHH: 1.57 ± 0.56%, CHG: 1.30 ± 0.61%, mean ± s.d.; electronic supplementary material, table S1) and significantly correlated (*r* = 0.45, *p* = 0.01). We did not find any correlation between non-CpG methylation and *avpr1a* expression level (*p* > 0.1), thus it is likely that our non-CpG methylation measurements merely reflect incomplete bisulfite conversion rather than true methylation. Based on this, we can estimate the rates of bisulfite conversion in our target by calculating 1 − %non-CpG methylation. We estimate our bisulfite conversion rate to range from 94.1% to 98.7% (97.1 ± 1.0%, mean ± s.d.), which is consistent with the conversion rate estimates from pyrosequencing quality controls.

### 3.3. Patterns of CpG methylation across *avpr1a* and among wild-caught voles

We partitioned the bis-seq target with CpGi, and prior gene annotations into sequence features we label as CpGi.1, Promoter, CpGi.2a, CpGi.2b, CpGi.2c, Intron and Enhancer (see Material and methods). Our bis-seq measurements show that DNA methylation varies greatly along the *avpr1a* locus and can differ dramatically among gene features (figure 2*c*). Average DNA methylation was low in CpGi.1 (7.5 ± 13.9%, mean ± s.d.) and promoter (1.8 ± 0.6%, mean ± s.d.). However, along CpGi.2, DNA methylation appears much more variable. Average DNA methylation was low in the 5′ end of CpGi.2, which includes CpGi.2a (1.2 ± 1.3%, mean ± s.d.) and CpGi.2b (5.1 ± 6.4%, mean ± s.d.), but increased within CpGi.2c (44.6 ± 26.6%, mean ± s.d.) and toward the exon1–intron boundary. The increase in methylation at the border of CpGi.2b and CpGi.2c coincides with a mouse (*Mus musculus*) transcript start peak from cap analysis gene expression data (electronic supplementary material, figure S1; [35]), suggesting this region may be involved in an unknown transcriptional function. Average CpG methylation was high throughout the intron (71.6 ± 18.8%, mean ± s.d.) and intron enhancer (72.5 ± 17.0%, mean ± s.d.; figure 2*c*).

DNA methylation did not vary drastically among individuals at CpG sites within the CpGi.1, promoter, CpGi.2a and CpGi.2b. However, CpGi regions with higher average DNA methylation (i.e. CpGi.2c) and CpGi-shore features—such as the intron and putative enhancer—exhibited high inter-individual variation. Individual differences in methylation at the 5′ end of CpGi.2c exist in the absence of CpG polymorphism. However, many CpG sites in the intron and the enhancer are polymorphic and it seems that methylation variation in this region was driven by genotype differences among individuals (figure 2*c*).

### 3.4. CpG co-methylation across the *avpr1a* locus

**Figure 3.**
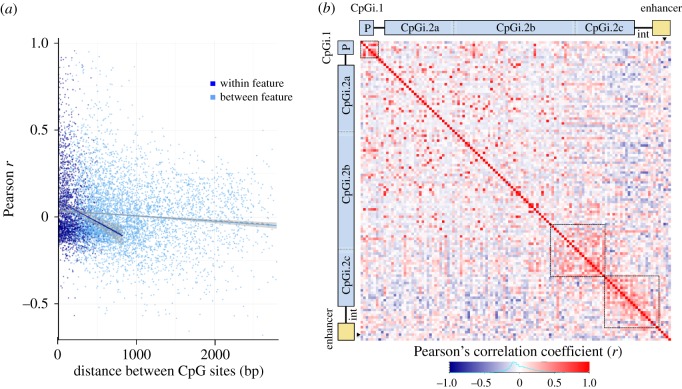
Patterns of CpG co-methylation across *avpr1a*. (*a*) Co-methylation measures (Pearson's correlation coefficient, *r*) are plotted against distance of CpG pairs within (dark blue, *r* = −0.14, *p* < 0.0001) and between gene features (light blue, *r* = −0.09, *p* < 0.0001). (*b*) Co-methylation between 122 fixed and polymorphic CpG sites depicted in a heatmap. Corresponding gene features are schematized on top and left, position of polyCpG 2170 is marked by black triangles and abbreviations are as follows: P = promoter, int = intron). Three clusters of positive co-methylation are outlined by dashed lines.

We used our bis-seq data to examine the correlation of DNA methylation (co-methylation) between pairs of CpG sites across *avpr1a*. In general, the strongest methylation correlations (|*r*| > 0.5) were found between close CpG pairs (less than 1 kb; figure 3*a*). We found a negative correlation between co-methylation and CpG distance. This correlation was significant for both same gene-feature (*r* = −0.14, *p* < 10 × 10^−8^) and between gene-feature co-methylation (*r* = −0.09, *p* < 10 × 10^−11^). However, the correlation was stronger among CpG pairs within the same feature (distance × CpG feature position *p* < 10 × 10^−7^; figure 3*a*).

Patterns of co-methylation were heterogeneous across the bis-seq target, as evident by three clusters of high positive correlation (figure 3*b*). The first co-methylation cluster was found upstream of the *avpr1a* TSS, within the CpGi.1 and promoter. The second cluster was located at the 3′ end of the second CpG island and included some CpGs within CpGi.2b and CpG1.2c. The third cluster was found on the exon–intron boundary and included CpGs from both CpG.2c and the intron, but not the intron enhancer. CpGs in the latter cluster showed overall negative methylation correlation with many other CpGs located upstream of the TSS (i.e. CpGi.1 and promoter) and the 5′ side of the first exon (CpGi.2a and parts of CpGi.2b; figure 3*b*).

### 3.5. Methylation and V1aR abundance in the retrosplenial cortex

**Figure 4.**
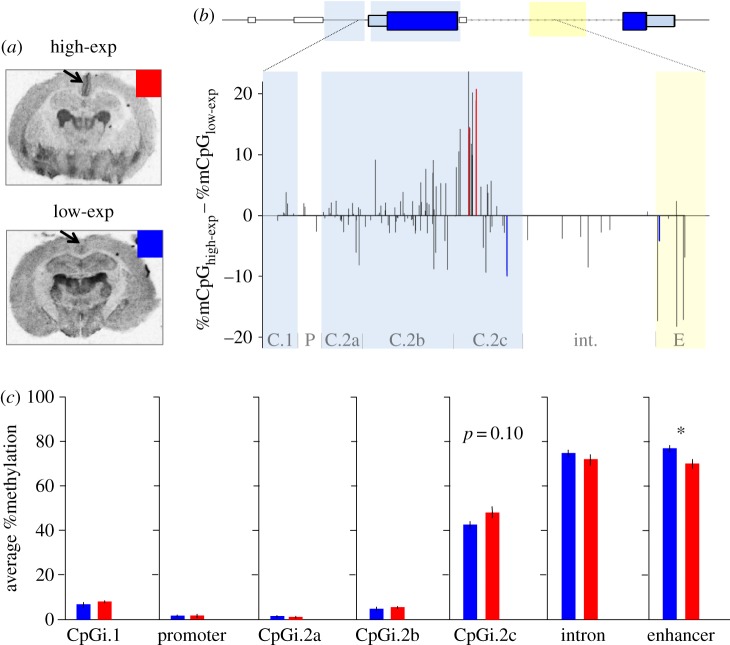
Relationship between *avpr1a* DNA methylation and RSC-V1aR. (*a*) Autoradiograms of V1aR show dramatic variation in RSC of wild-caught voles. Median split divided individuals into high-exp (red) and low-exp (blue). (*b*) CpG methylation differences between high-exp and low-exp at 122 CpG sites. Gene features marked on bottom of graph (abbreviations are as follows: C.1 = CpGi.1, P = Promoter, C.2a = CpGi.2a, C.2b = CpGi.2b, C.2c = CpGi.2c, int. = Intron, E = Enhancer). Red bars denote methylation higher among high-exp, blue bars lower methylation among high-exp (*t*-test, *p* ≤ 0.05). (*c*) Average feature methylation in high-exp (red) and low-exp (blue) individuals. Bars are means ± s.e. *p ≤ 0.05.

We observed substantial variation in the abundance of RSC-V1aR among our wild-caught voles (figure 4*a*). To examine the relationship between RSC-V1aR abundance and *avpr1a* methylation, we split individuals at the median value of RSC-V1aR (median = 5669.5 dpm mg^−1^ TE) into high-expressing (high-exp) and low-expressing (low-exp; figure 4*a*). We compared DNA methylation between the high-exp and low-exp wild voles at individual CpG sites and gene features.

In our single CpG comparisons, first we averaged DNA methylation of all individuals within high- and low-exp animals at each of the 122 CpG sites and calculated their difference (figure 4*b*). Methylation differences were generally small (less than 10%), but many CpGs in the 3′ end of CpG.i2 showed higher methylation in high-exp animals. These sites seem to correspond to cluster 2 in our co-methylation analysis. By contrast, CpGs within the enhancer showed lower methylation in high-exp animals. Using *t*-tests, we found four CpG sites with different methylation between high- and low-expressing animals (*p* < 0.05; figure 4*b*); however, none survived false discovery rate corrections (adjusted *p* > 0.1, [31]). Three of these CpGs were in CpGi.2c, and one was in the enhancer region.

Examining average levels of methylation across features, we found a significant difference in DNA methylation between high- and low-exp animals in the putative enhancer (high-exp: 70.10 ± 2.07%, low-exp: 77.02 ± 1.40%, mean ± s.d., *p* = 0.01; figure 4*c*). Average CpGi.2c methylation was higher in the high-expressing animals (high-exp: 47.94 ± 2.72%, low-exp: 42.60 ± 1.60%, mean ± s.e.; figure 4*c*), but this difference was not statistically significant (*p* = 0.10). None of the other gene features exhibited methylation differences between high- and low-exp animals (*p* > 0.10; figure 4*c*).

### 3.6. *avpr1a* genotypes and the putative intron enhancer

**Figure 5.**
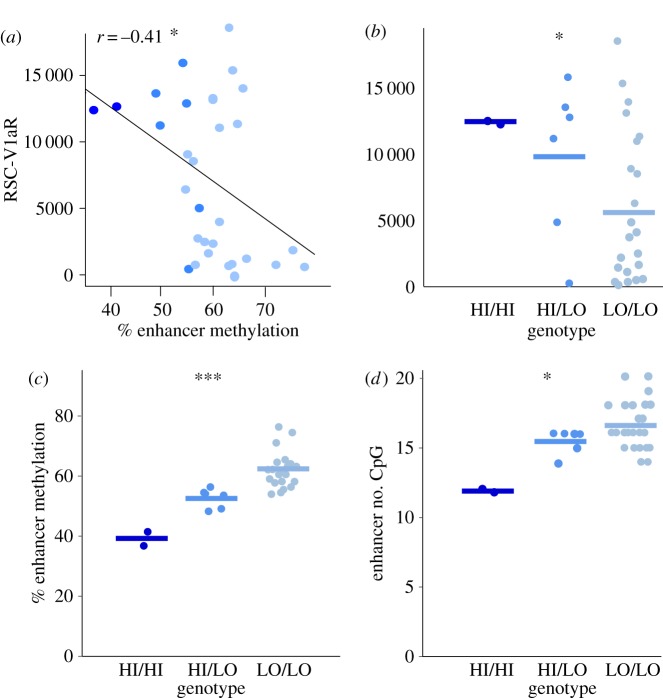
*avpr1a* genotype differences in RSC-V1aR, enhancer methylation and CpG density. (*a*) DNA methylation in the enhancer is negatively correlated with RSC-V1aR abundance. (*b*) *avpr1a* genotypes (HI/HI, HI/LO and LO/LO) differ in RSC-V1aR [24] and (*c*) average enhancer methylation. (*d*) CpG count within enhancer correlates with *avpr1a* genotype. All bars are means. *p ≤ 0.05, ***p ≤ 0.001.

Average %DNA methylation in the putative intron enhancer was negatively associated with RSC-V1aR among wild voles (*r* = −0.41, *p* = 0.03; figure 5*a*). As previously reported [24], we found 24 LO/LO, six heterozygous HI/LO and two HI/HI individuals, and these genotypes differ in RSC-V1aR abundance (ANOVA, *F* = 4.99, *p* = 0.03; figure 5*b*, see also [24]). Here, we find that these individuals also differ in average enhancer methylation (HI/HI 39.4 ± 3.2%, HI/LO 55.4 ± 4.3%, LO/LO 63.3 ± 6.4%, mean ± s.d.; ANOVA, *F* = 20.23, *p* < 0.0001; figure 5*c*). Sequence differences between the HI and LO allele involve enhancer polyCpGs, which leads to genotype differences in numbers of CpG sites within the putative enhancer (HI/HI: 12.0 ± 0.0, HI/LO: 15.5 ± 0.3, LO/LO: 16.6 ± 0.4, mean #CpG ± s.d.; Kendall's *τ* = 0.38, *p* = 0.016; figure 5*d*) but not across the whole *avpr1a* locus (HI/HI: 356.5 ± 0.7, HI/LO: 364.2 ± 3.4, LO/LO: 364.2 ± 5.8, mean #CpG ± s.d.; Kendall's *τ* = 0.17, *p* = 0.27; data not shown).

### 3.7. CpG polymorphisms

Among our wild voles, we found 30 polyCpGs across the *avpr1a* locus (electronic supplementary material, table S2). We used the bi-allelic polyCpGs (*n* = 29) to examine the frequency of each polyCpG variant. The frequency distribution of polyCpG variants was highly divergent from null expectations ($x_{\left(5,n=29\right)}^{2}=30.37$, *p* < 0.0001; table 2). More than half of the variants (79.2%) were G/A or C/T polymorphisms (table 2), which is consistent with the expected prevalence of methylation-induced de-amination mutations and previous genome-wide characterizations of polyCpG frequencies [18].

**Table 2.** Frequency of polyCpG variants across the *avpr1a* locus.

CpG polymorphism	frequency
CpG/CpA	44.8% (13/29)
CpG/CpC	3.4% (1/29)
CpG/CpT	7.0% (2/29)
CpG/ApG	7.0% (2/29)
CpG/GpG	3.4% (1/29)
CpG/TpG	34.4% (10/29)

**Figure 6.**
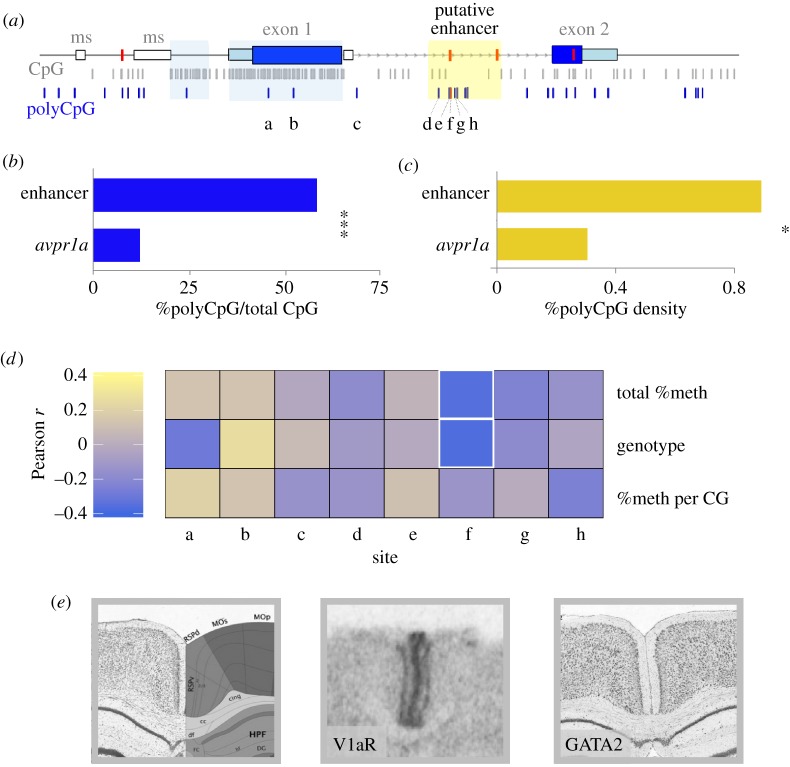
Distribution of polyCpGs and their sequence-specific associations with RSC-V1aR. (*a*) Distribution of fixed (grey) and polymorphic CpGs (blue) along *avpr1a* locus. The four linked SNPs that define HI and LO alleles are marked with red bars on the locus. The eight polyCpGs covered in bis-seq assay are labelled *a–h* (modified from [24]). (*b*) Per cent polyCpGs/total CpG in the enhancer is compared with the rest of the locus. (*c*) Density of polyCpGs (polyCpGs per 100 bp) within enhancer compared with rest of locus. (*d*) For each polyCpG Pearson's correlation coefficient is calculated between RSC-V1aR abundance and total % DNA methylation (*top*), genotype (*middle*), and %methylation per CpG allele (*bottom*). Cells with *p* ≤ 0.05 are outlined with a white border. (*e*) *Left*, Nissl image and atlas of mouse brain at the retrosplenial area (RSP). *Centre,* prairie vole autoradiogram shows V1aR abundance at the retrosplenial cortex (RSC). *Right,* antisense RNA *in situ* staining shows expression of GATA2 in the retrosplenial area of mouse (Image credit: Allen Institute. © 2015 Allen Institute for Brain Science. Allen Mouse Brain Atlas. Available from: http://mouse.brain-map.org/gene/show/14237). *p ≤ 0.05 and ***p ≤ 0.001.

PolyCpGs were also non-homogeneously distributed across *avpr1a*. The two CpG islands, which together accommodate 72.1% of all the fixed *avpr1a* CpGs, only hold three polyCpGs; a significantly lower polymorphisms rate compared with the rest of the locus (Fisher's exact, *p* < 0.0001; figure 6*a*). By contrast, the 786 bp enhancer has seven polyCpGs and five fixed CpGs. The remaining 7530 bp of the *avpr1a* locus holds 23 polyCpGs and 167 fixed CpG sites (figure 6*a*). Thus, a larger fraction of CpG sites are polymorphic within the enhancer than across the rest of the locus (58.3% versus 12.1%, Fisher's exact, *p* = 0.0004; figure 6*b*). Similarly, polyCpG density is higher in the enhancer compared with the rest of the locus (0.89% versus 0.31%, Fisher's exact, *p* = 0.02; figure 6*c*).

In our bis-seq assay, we captured eight of the *avpr1a* polyCpG sites: five located within the putative intron enhancer and one in each of the CpGi.2b, CpGi.2c and intron features. We found that *total %DNA methylation* (*r* = −0.31, *p* = 0.052) and *genotype* (*r* = −0.32, *p* = 0.045) at polyCpG 2170 were associated with RSC-V1aR (figure 6*d*). This polyCpG is one of the SNPs that define the HI and LO allele in both laboratory-reared and wild-caught animals [24]. The seven remaining polyCpG sites did not predict individual differences in RSC-V1aR (*p* > 0.10).

Transcription factor affinity (sTRAP) analysis at polyCpG 2170 provided a list of candidate transcriptions factors predicted to bind to this sequence. These transcription factors are expected to show a highly different affinity between the HI and LO allele (table 3). Examination of the Allen Brain Atlas (figure 6*e*; [32]) revealed that at least one of these transcription factors (GATA2) had strong expression in the mouse retrosplenial area. GATA2 binding is predicted to be much stronger to the LO allele sequence than to the HI allele sequence. Not all of the factors exhibited clear evidence of expression in the mouse RSC based on the Allen Brain Atlas, but the atlas is descriptive, and negative data are inconclusive.

**Table 3.** Transcription factor affinity for the HI and LO alleles at polyCpG 2170.

difference log(*p*)	HI *p*-value	LO *p*-value	matrix ID	matrix name	transcription factor
−2.47	0.803	<0.00273	M00075	V$GATA1_01	GATA binding protein 1
−1.08	0.171	0.0143	M01082	V$BRCA_01	BRCA
−1.03	0.154	0.0145	M00076	V$GATA2_01	GATA binding protein 2

## 4. Discussion

In nature, individual differences arise as genetic and epigenetic forces interact to shape gene expression, cellular processes and organismal phenotypes. In this study, we explore DNA methylation and CpG distribution at *avpr1a,* the locus encoding the vasopressin 1a receptor (V1aR). We characterized CpG distribution across approximately 8 kb of the *avpr1a* locus and found dramatic variation in CpG density (figure 2*a*). The highest CpG density was found in two CpG islands that flanked the TSS (figure 2*a*). Next, we used high-throughput sequencing techniques and natural genetic variation among 30 wild prairie voles to examine the significance of DNA methylation and polymorphic CpGs (polyCpGs) in shaping cortical *avpr1a* expression associated with complex spatial and sexual behaviours.

We used a targeted bis-seq approach to characterize DNA methylation at 122 CpG sites across approximately 3 kb of the *avpr1a* locus. Within the intron, we showed high correlation between methylation measures obtained by traditional pyrosequencing and our targeted bis-seq approach (figure 2*b*). The correlation was better among polymorphic intron CpGs compared with the fixed sites, but fixed intron CpGs had uniformly high levels of methylation. It also appears that bis-seq methylation measures were a little higher than pyrosequencing measures. The exact reason for this is not known, but we speculate that the higher GC content of methylated fragments may make them easier to amplify during the bis-seq library preparation. The main discrepancy, however, is at the 3′ end of the first pyrosequencing assay (CpG 2113, figure 2*b*), where pyrosequencing results are more error-prone [36]. If so, we expect the bis-seq measures to be more accurate. Another issue worth noting is that our methylation measures have been collected and averaged over multiple cell types from RSC dissections. While this is much better than whole brain or proxy tissue analyses, averaging across multiple cell types suggests a measure of caution. Nevertheless, on balance our technical validations suggest the targeted bis-seq approach is a useful means for exploring methylation variation across a targeted locus and among multiple individuals.

We found dramatic methylation changes across *avpr1a* gene features (figure 2*c*). We observed low methylation at features with high CpG density (i.e. CpG islands and promoter), and substantially higher levels of methylation as CpG density declined near the end of the first exon and into the intron. Consistent with genome-wide studies [37,38], our co-methylation analysis revealed that CpG methylation was correlated at neighbouring CpG sites (less than 1 kb), especially between CpGs in the same gene feature (figure 3*a*). Stronger co-methylation between CpGs within features suggests these labels capture meaningful dimensions of epigenetic regulation across individuals.

Remarkably, examination of V1aR abundance in the retrosplenial cortex (RSC-V1aR) revealed that the methylation status of the *avpr1a* promoter did not predict gene expression (figure 4*c*), because the *avpr1a* promoter remains uniformly unmethylated, even in individuals with low V1aR abundance. This is consistent with recent reports from genome-wide studies of mammalian brains [39] and multiple cell lines [16,40], which find that CpG-rich promoters are often unmethylated. Indeed, recent work inserting randomized sequences into the mouse genome reveals that sequences with high GC content and high CpG abundance are sufficient to prevent CpG methylation [41]. By contrast, work in behavioural epigenetics often focuses more narrowly on individual differences in promoter methylation. For CpG-rich promoters, a lack of methylation seems to be necessary but not sufficient for gene expression. These results emphasize the need to look beyond promoter methylation to interpret epigenetic variation, either in a cell line or among individuals in the wild.

In contrast with the *avpr1a* promoter, gene features located in a neighbouring CpGi-shore had high methylation levels. Average methylation sharply increased around the first exon–intron boundary and remained high (more than 50%) throughout the intron and enhancer (figure 2*c*). Sharp methylation transitions at the exon–intron boundary are thought to serve as a signal for regulation of transcription and mRNA splicing [13]. Interestingly, our analysis revealed heterogeneous patterns of co-methylation across the *avpr1a* locus, including two clusters of co-methylated CpGs around the exon–intron boundary (figure 3*b*), suggesting these groups of CpGs are coherent regulatory units. We also noted a trend towards higher methylation at CpGs immediately upstream of the exon–intron border in animals with elevated *avpr1a* expression (figure 4*b*). These patterns of coding sequence methylation are all consistent with the hypothesized role of DNA methylation in the specification and splicing of exons during transcription [13].

In general, intron CpG sites were highly methylated and poorly predictive of RSC-V1aR abundance. However, within a previously identified putative intron enhancer [24], methylation levels were both more varied and predictive. We found that wild voles with high abundance of RSC-V1aR have lower methylation within the intron enhancer (figures 4*b*,*c* and 5*a*). The specificity of this relationship suggests that the lack of CpG methylation at the *avpr1a* promoter may be permissive, while methylation of the intron enhancer may inhibit RSC-V1aR expression. This is consistent with recent studies suggesting genes with CpG islands in the promoter have reliably low levels of methylation, while regulatory elements with low to intermediate CpG density are more likely to exhibit individual or tissue-specific methylation and regulation [42,43]. Similarly, intron enhancers have been documented for a variety of genes [44,45], and loss of DNA methylation can activate such enhancers [46].

We recently reported two *avpr1a* alleles that strongly predicted RSC-V1aR abundance and enhancer methylation in laboratory-reared animals [24]. These HI and LO alleles were defined by four highly linked SNPs across the *avpr1a* locus [24]. Among our wild-caught voles, HI and LO genotypes again show different levels of RSC-V1aR and enhancer methylation (figure 5*b*,*c*; also [24]). We find that the HI and LO alleles of wild voles also differ in the total number of enhancer CpGs (figure 5*d*). Moreover, polyCpGs are significantly more common in the intron enhancer than in the rest of the locus (figure 6*a*–*c*). This uneven distribution of polyCpGs suggests that they may be playing a functional role in regulating expression, perhaps by altering the sensitivity of the enhancer to developmental methylation. We next examined alternative models of how polyCpGs could influence V1aR expression by exploring different measures of methylation at each polyCpG site within our bis-seq target.

Polymorphic CpGs in the *avpr1a* putative enhancer could influence RSC-V1aR variation by overall changes in CpG and methylation density, by disrupting transcription factor binding sites, or by some more complex combination of the two (figure 1). If a given polyCpG were influencing expression by contributing to overall levels of methylation, this may result in a correlation between *total %DNA methylation* and expression (figure 1*a*). By contrast, if a CpG polymorphism influenced expression because only one of the alleles was recognized by a transcription factor, then we would expect to see an association between expression and *genotype* (figure 1*b*). Lastly, if a methylation-sensitive transcription factor binds to the CpG allele at this site, we would expect to find a correlation between expression and the proportion of methylated CpG alleles (*%methylation per CpG*; figure 1*c*). In seven of eight polyCpGs examined across our bis-seq target, we found no associations between methylation or genotype and expression—these polyCpGs do not seem to shape transcription factor binding sites. They might, however, still contribute in aggregate to regulation through overall methylation. Interestingly, in one polymorphism (polyCpG 2170) we found that both *total %DNA methylation* and *genotype* predicted RSC-V1aR (figure 6*d*). This site is unique in being both a polyCpG and one of the SNPs that define HI and LO alleles associated with genetic variation in RSC-V1aR.

One interpretation of the genotype effect at polyCpG 2170 (figure 6*d*) is that it is simply a by-product of its strong linkage to other SNPs of the HI and LO alleles. These other SNPs are not within the bis-seq target, nor are they polyCpGs, but may nevertheless influence expression. This does not, however, preclude a causal role for polyCpG 2170, and the fact that both its genotype and methylation status predict expression suggests it may be a direct contributor. One plausible mechanism is that a sequence-sensitive transcription factor may bind to this site (figure 1*b*). Based on published position weight matrices [30], we identified three transcription factors that show allele-specific binding to the sequence containing this SNP (table 3). Interestingly, all three transcription factors showed substantially higher affinity for the LO allele, but none favoured the HI allele. At least one of these transcription factors, GATA2, is expressed in the mouse RSC (figure 6*e*; [32]). While GATA2 often activates gene expression, it has also been shown to silence expression [47]. Thus GATA2, or some other transcription factor, could directly silence the LO *avpr1a* allele in the vole RSC. In this scenario, the genotype differences in overall enhancer methylation may actually be a downstream consequence of transcription factor-induced silencing.

An alternative interpretation of our findings is that methylation at polyCpG 2170, possibly in aggregate with methylation at other weakly linked enhancer polyCpGs, could suppress the LO allele by attracting methyl-binding proteins such as MeCP2 [16]. Like the first scenario, it suggests a complex interaction between genetic variation and methylation. Unlike the first scenario, however, this mechanism is not sequence-specific, as it does not depend on the exact sequence context of the enhancer polyCpGs. Unfortunately, these two interpretations cannot be distinguished with our current data. Approaches that characterize transcription factor binding to DNA *in vivo*, or that manipulate CpG density while leaving a focal SNP intact, could clarify the nature this interaction. In either case, our data demonstrate that attempts to link DNA sequence, methylation status and gene expression might do well to focus on enhancers rather than promoters. Such studies will be critical to understanding how genetic variation interacts with developmental environment to produce individual differences in complex behaviours.

In conclusion, we have used modern molecular tools to characterize how CpG distribution and polymorphism predict methylation and expression of the *avpr1a* locus in the brain of wild prairie voles. We find that a targeted bis-seq approach recapitulates traditional pyrosequencing methods, but allows characterization of a larger set of CpG sites. We find that the regulatory effects of *avpr1a* methylation are highly dependent on genetic context: enhancer methylation was associated with low expression while promoter status was not; similarly, methylation in the gene-body may shape transcription and splicing of *avpr1a*. Most polymorphic CpGs do not contribute to *avpr1a* expression by altering transcription factor binding sites. Rather, allelic differences in methylation or transcription factor binding at polyCpG 2170, seem to shape the effects of the intron enhancer on cortical V1aR and its downstream behaviours. Future studies that target candidate transcription factors, or that modify DNA sequence and/or methylation, will be required to determine the precise mechanisms by which sequence variation influences *avpr1a* expression. Overall, our results illustrate some of the complex ways that genetic and epigenetic variation can interact to shape brain and behaviour in the wild. Such studies will prove critical to our understanding of plasticity, adaptation and evolution.

## Supplementary Material

Figure S1. CAGE data reveal the 5’ boundary of transcripts along the bis-seq target. A) Cap analysis gene expression (CAGE) sequencing reads from the FANTOM5 project (35) are viewed at the mouse (mm10) avpr1a (http://genome.ucsc.edu), within a region homologous to the vole bis-seq target. Significant CAGE peaks are marked by black bars on the bottom of track. B) Modified figures from Figure 2, show CpG density and methylation across bis-seq gene-features. Gene-feature borders are depicted by dashed lines.

## Supplementary Material

Table S1. Targete bis-seq metadata on wild-caught prairie voles.

## Supplementary Material

Table S2. Position and sequence polymorphism at polyCpG sites across the avpr1a locus. SNP positions are based on avpr1a reference AF069304.2 (NCBI) and abbreviations are as follow: CpGi = CpG island, CDS = coding sequence, UTR = untranslated region.

## Data accessibility

Protein and bis-seq DNA methylation metadata are provided in electronic supplementary material, table S1. Position and sequence polymorphism for all 30 *avpr1a* polyCpGs are presented in electronic supplementary material, table S2. Raw targeted bis-seq reads are available in the Dryad Digital Repository: http://dx.doi.org/10.5061/dryad.f8d4r [48].
