# Supplementary figures and images for: Methylation of avpr1a in the cortex of wild prairie voles: effects of CpG position and polymorphism
Source: R Soc Open Sci. 2017 Jan 18;4(1):160646. doi: 10.1098/rsos.160646 (PMC5319330; doi:10.1098/rsos.160646)

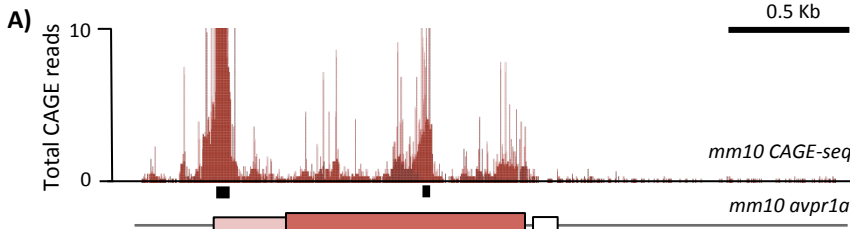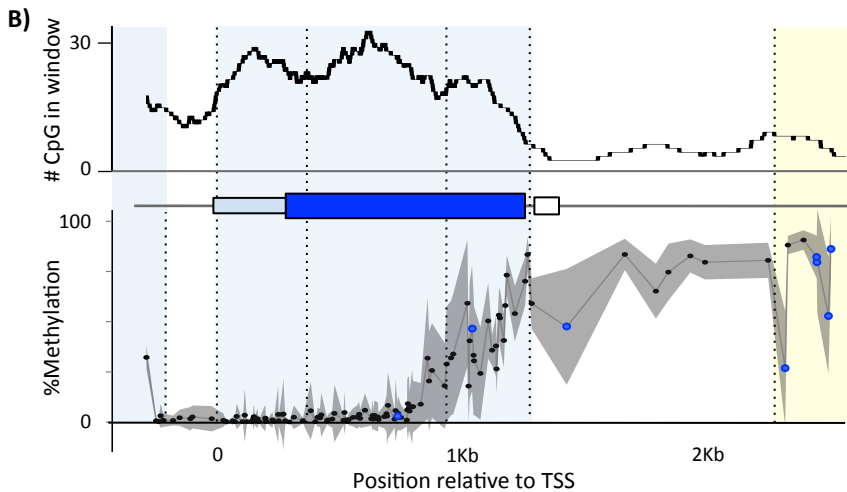

Supplement: Figure S1. CAGE data reveal the 5’ boundary of transcripts along the bis-seq target. A) Cap analysis gene expression (CAGE) sequencing reads from the FANTOM5 project (35) are viewed at the mouse (mm10) avpr1a (http://genome.ucsc.edu), within a region homologous to the vole bis-seq target. Significan [file rsos160646supp1.pdf]
